# Bone Mineral Density Assessment by Quantitative Computed Tomography in Glucocorticoid-Treated Boys With Duchenne Muscular Dystrophy: A Linear Mixed-Effects Modeling Approach

**DOI:** 10.3389/fendo.2022.860413

**Published:** 2022-03-23

**Authors:** Chuan Liu, Dan-Dan Yang, Lu Zhang, Xian-Gao Lei, Feng-Lin Jia, Yi Liao, Xi-Jian Chen, Gang Ning, Wen Luo, Hai-Bo Qu

**Affiliations:** ^1^ Department of Radiology, Key Laboratory of Obstetric and Gynecologic and Pediatric Diseases and Birth Defects of Ministry of Education, West China Second University Hospital, Sichuan University, Chengdu, China; ^2^ Department of Radiology, The Third People’s Hospital of Chengdu, Chengdu, China; ^3^ Department of Radiology, Chengdu Qingbaijiang District People’s Hospital, Chengdu, China

**Keywords:** Duchenne muscular dystrophy, osteoporosis, bone mineral density, glucocorticoids, quantitative computed tomography

## Abstract

**Objective:**

Boys with Duchenne muscular dystrophy (DMD) are at risk of bone damage and low bone mineral density (BMD). The aim of the study is to examine lumbar BMD values measured by QCT and identify the factors associated with BMD loss using a multilevel mixed-effects model.

**Methods:**

Lumbar BMD was evaluated by quantitative computed tomography (QCT) at diagnosis, 1 and 2 years follow up in patients with DMD who were treated with GC. Demographic data, functional activity scores (FMSs), laboratory parameters and steroid use were recorded. A multilevel mixed-effects model was used to analyze BMD loss.

**Results:**

Nineteen patients with DMD who had a total of sixty complete records between January 2018 and October 2021 were retrospectively analyzed. At baseline, 15.8% of patients (3/19) had low lumbar BMD (Z score ≤ −2), and the mean BMD Z score on QCT was -0.85 (SD 1.32). The mean BMD Z score at 1 and 2 years postbaseline decreased to -1.56 (SD 1.62) and -2.02 (SD 1.36), respectively. In our model, BMD Z score loss was associated with age (β=-0.358, p=0.0003) and FMS (β=-0.454, p=0.031). Cumulative GC exposure and serum levels of calcium, phosphorus, 25(OH)-vitamin D and creatinine kinase did not independently predict BMD loss.

**Conclusions:**

This study demonstrates that in DMD patients, lumbar BMD decreased gradually and progressively. Age and FMS are the main contributors to BMD loss in boys with DMD. Early recognition of risk factors associated with BMD loss may facilitate the development of strategies to optimize bone health.

## Introduction

Duchenne muscular dystrophy (DMD) is an X-linked disorder that is associated with progressive muscle wasting and weakness, occurring in 1 of 3000-5000 live male births (1, 2). Boys with DMD usually present symptoms before six years of age, lose independent ambulation during the teenage years and are life-limited by the third decade of life, usually due to cardiorespiratory compromise (3). Low bone mineral density (BMD) is a common feature in patients with DMD and is associated with poor clinical outcomes and quality of life. Osteoporotic fractures may occur during normal activities of daily living in these patients, with a reported incidence of 21-44% (4, 5).

Although recent DMD care guidelines recommend serial spine radiographs to assess changes in spine morphology to monitor and diagnosis osteoporosis (6), BMD values still play a critical role in determining the overall trajectory of bone health, regardless of whether a patient has bone fragility. Baseline and annual dual-energy X-ray absorptiometry (DXA) scans for DMD patients have also been suggested (6). However, there are limitations of DXA in evaluating BMD that should be noted. Several studies have shown that spine deformities or anatomical changes can cause inaccuracies in BMD measurements made with DXA (7–9). Therefore, BMD Z scores adjusted for age-matched, height, bone age or bone size have been used to more accurately estimate the actual BMD and evaluate bone health in boys with DMD (10–14). In contrast, quantitative computed tomography (QCT) is not subject to these limitations because it is able to correct the scoliosis curves and directly measure the true volumetric BMD at lumbar trabecular bone. In addition, trabecular bone tends to be more metabolically active than cortical bone and responds quickly to treatment (15). To our knowledge, only one study about QCT-based BMD data in boys with DMD was published in 2020, and it showed that QCT markedly increased the diagnostic rate of osteoporosis compared to DXA (16).

Factors negatively affecting bone health in DMD patients include progressive muscular weakness, loss of weight bearing activity and potent osteotoxicity of long-term glucocorticoid (GC) therapy (17). To date, GC therapy is the only disease-modifying therapy. However, prolonged use of GC predisposes patients to osteoporosis by increasing bone resorption, decreasing bone formation and growth and delaying puberty (18, 19). On the other hand, mechanical stimulation may play a vital role in stimulating bone growth (20, 21). Furthermore, low levels of vitamin D cause abnormalities in osteoblast function and imbalances in calcium metabolism and can worsen this process. However, to our knowledge, which of these factors has the greatest impact on BMD loss in boys with DMD has not been well described.

Therefore, this study aimed to examine lumbar BMD values measured by QCT and identify the factors associated with BMD loss using a multilevel mixed-effects model, which might be helpful in developing strategies to optimize bone health and decrease the risk of fragility fractures in DMD.

## Materials and Methods

### Study Participants

We conducted a retrospective longitudinal study using data from the electronic medical records of patients with DMD at West China Second University Hospital from January 2018 to October 2021. A total of forty-two boys with DMD were confirmed by means of genetic testing and/or muscle biopsy during this period. Boys were excluded if they were not taking glucocorticoid therapy. Additionally, patients who were lost to follow-up or did not have BMD information were also excluded. Nineteen boys were selected who had undergone annual “bone health” assessments during at least 2 consecutive follow-ups irrespective of their age (i.e., they had at least 2 years follow up). This study was approved by the Institutional Review Board of West China Second University Hospital. (IRB# 20200021gc).

### Data Collection

All data were collected from the electronic medical records, including age, height, weight, functional activity scores (FMSs), laboratory parameters, status of steroid use, and BMD values at each clinic visit. The results of laboratory tests, including serum levels of calcium, phosphorus, 25(OH)-vitamin D, creatinine kinase (CK), creatine kinase isoenzyme (CK-MB) and intact parathyroid hormone (PTH), were recorded. The FMS reflecting their functional activity level was defined by Swinyard and Deaver’s 8-grade scale (22).

In GC-treated boys with DMD, the initial dose was usually given in the form of either prednisolone or deflazacort at 0.75 mg/kg/d or 0.9 mg/kg/d, respectively, and then the dose was increased according to body mass. At the same time, once the hormone was taken, the children were given oral vitamin D3 (400 IU/d) and elemental calcium (400 mg/d) in the form of dietary supplements. The conditions of GC use, including age at initiation of GC use, daily dose and length of treatment, were also recorded. The cumulative GC dose was calculated using information recorded in the subjects’ medical records.

### QCT Scanning and BMD Measurement Procedures

We used a Neusoft 128-slice helical CT scanner (NeuViz128, China) to acquire CT images of the lumbar spine (L1-L3; 120 kV, 70 mAs, 3-mm slice thickness). Quality control was ensured throughout the study through daily calibration and cross calibration with the European spine phantom (ESP-145) on 10 repeated scans acquired following a prescribed QCT scanning protocol. The quality assurance (QA) results showed that the ESP volumetric BMD measured at our center differed by less than 5 mg/cm^3^ on average.

**Figure 1:**
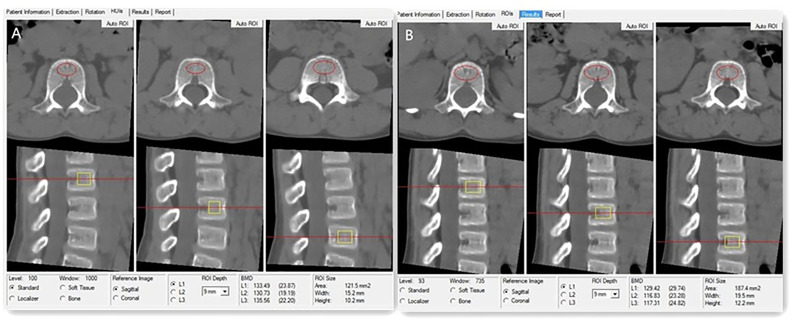
**(A)** Images for a 7-year-old boy with DMD who has been treated with GC for 2 years. The measurements of L1, L2, and L3 vertebral trabecular volumetric bone mineral density (BMD) are shown. The BMD of L1, L2, and L3 is 133.49 mg/cm^3^, 130.73 mg/cm^3^, and 135.56 mg/cm^3^, respectively; the average lumbar volumetric BMD is 133.26 mg/cm3, and the Z score is -1.55. **(B)** The same boy with DMD was followed up after 1 year of GC therapy. The measurements of L1, L2, and L3 vertebral trabecular volumetric bone mineral density (BMD) are shown; the BMD of L1, L2, and L3 is 128.42 mg/cm^3^, 116.83 mg/cm^3^, and 117.31 mg/cm^3^, respectively. The average lumbar volumetric BMD is 121.19 mg/cm3, and the Z score is -1.97.

Asynchronous BMD calibration in combination with QCT Pro analysis software (Mindways Software, Inc.) was used to obtain lumbar spine (L1–L3) trabecular volumetric BMD (mg/cm^3^) (**Figure 1**), as previously reported (16). References for vertebral BMD Z scores based on age and sex were provided by the manufacturer of the QCT software (Mindways Software) (23). A low BMD was defined as a Z score of ≤ -2.0 according to the current ISCD recommendations for children (15). All patients or their families provided written informed consent to undergo QCT scanning.

### Statistical Analysis

Statistical analysis was performed using SPSS software (version 22.0). A multilevel mixed effect model was used to analyze BMD loss. Level one was the patient’s relevant measurements at different time points, and level two was the patient. The outcome variable was BMD Z score. First, the intraclass correlation coefficient (ICC) was used to determine whether the dependent variable was significantly different among individual levels and consider the necessity of establishing a multilevel model. When high levels were significant, independent variables including age, serum calcium, phosphorus levels, 25(OH)-vitamin D, creatinine kinase (CK), FMS, and cumulative GC dose were added to the fixed-effect portion of the model. Interaction effects between each independent variable and time were also considered. If the interaction term was not statistically significant, it was omitted from the model. A two-tailed *p* value <0.05 was considered as statistically significant.

## Results

### Baseline and Follow-up Characteristics

Nineteen boys with GC-treated DMD who had a total of sixty complete records from January 2018 to October 2021 were included in this study. At baseline, the mean age was 8.58 ± 1.87 years, and the mean age at GC therapy initiation was 6.67 ± 2.19 years. Serum CK and CK-MB levels increased gradually with increasing follow-up time. The serum calcium levels were in the normal range in 19 patients, and only 1 patient had decreased levels of serum calcium at the second year of follow-up. The serum phosphorus levels decreased in 4 patients (21.05% decrease) at baseline, and serum phosphorus levels decreased in 7 and 8 patients after 1 and 2 years of follow-up, respectively. The levels of serum 25(OH)-vitamin D were in the range of deficiency, with a mean of 19.90 ± 4.28 ng/dL at baseline; 11 patients (57.89%) had vitamin D deficiency, and 8 patients (42.11%) had insufficiency. The mean levels of serum 25(OH)-vitamin D showed a decreasing tendency with increasing follow-up. The demographic and clinical characteristics at baseline and follow-up in patients with DMD are summarized in **Table 1**.

**Table 1:** Demographic and clinical characters at baseline and follow-up for patients with DMD.

Clinical variables	Baseline (n = 19)	Year 1 (n = 19)	Year 2 (n = 19)	Year 3 (n = 3)
Age (years)	8.58 ± 1.87	9.69 ± 2.06	11.15 ± 1.89	12.73 ± 2.08
BMI	18.25 ± 2.39	19.50 ± 3.94	19.88 ± 4.22	19.54 ± 3.53
Lumbar BMD (mg/cm^3^)	127.83 ± 38.49	123.90 ± 39.54	116.53 ± 38.58	115.43 ± 10.92
T value	-1.75 ± 1.36	-1.87 ± 1.38	-2.15 ± 1.47	-2.13 ± 0.52
Z score	-0.85 ± 1.32	-1.56 ± 1.62	-2.02 ± 1.36	-2.41 ± 0.51
≤−1.5	4 (21.1%)	9 (47.4%)	13 (63.2%)	3 (100.0%)
≤−2	3 (15.8%)	6 (31.6%)	6 (31.6%)	2 (66.7%)
Age started GC (years)	6.75 ± 2.34			
Duration (M)	18.39 ± 10.78	33.89 ± 10.58	44.78 ± 14.45	61.33 ± 15.28
Cumulative exposure (g)	5.79 ± 3.77	11.9 ± 4.21	16.81 ± 5.92	23.72 ± 2.98
Decreased of calcium	0 (0%)	0 (0%)	1 (5.26%)	0 (0%)
Decreased of phosphorus	4 (21.05%)	7 (36.84%)	8 (42.11%)	1 (33.33%)
CK-MB (U/L)	158.32 ± 94.48	108.64 ± 77.19	99.65 ± 74.09	79.47 ± 57.97
CK (x10^3^ UG/L)	15.81 ± 7.74	11.42 ± 5.82	12.87 ± 9.06	10.72 ± 2.72
25 (OH)-Vitamin D (ng/dL)	19.90 ± 4.28	19.14 ± 3.99	18.30 ± 4.74	15.17 ± 1.55
20-30 ng/dL	8 (42.11%)	6 (31.58%)	6 (31.58%)	0 (0%)
≤20 ng/dL	11 (57.89%)	13 (68.42%)	13 (68.42%)	3 (100.00%)
FMS	1.37 ± 0.68	1.37 ± 0.60	1.89 ± 0.94	1.67 ± 1.16
FMS>1	5 (26.32%)	6 (31.58%)	10 (52.63%)	1 (33.33%)

Data are presented as mean means ± standard deviation or n (%).

DMD, duchenne muscular dystrophy; BMI, body mass index; BMD, bone mineral density; GC, glucocorticoid; CK-MB, creatine kinase isoenzyme; CK, creatinine kinase; FMS, functional activity score.

### Assessment of Lumbar BMD

**Figure 2:**
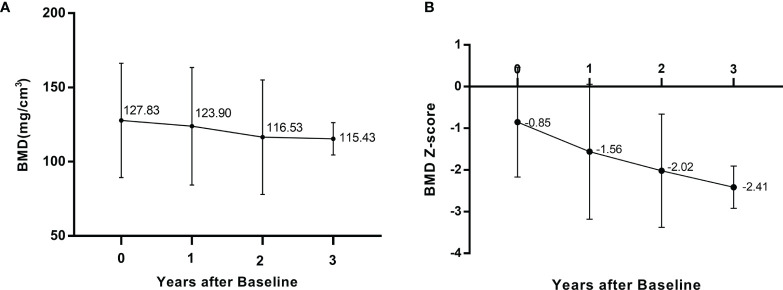
Longitudinal changes lumbar intrabecular BMD **(A)** and lumbar BMD Z score **(B)** at baseline and follow-up.

At baseline, QCT scans showed reduced lumbar BMD (Z score ≤ −2) in 3 (15.8%) patients and normal lumbar BMD (Z score >−2) in 16 (84.2%) patients; the mean volumetric BMD of the lumbar spine was 127.83 ± 38.49 mg/cm^3^, and the corresponding BMD Z score was -0.85 ± 1.32, ranging from 1.68 to -3.34. During follow-up, the BMD Z score at year 1 and year 2 postbaseline decreased to a mean of -1.56 (SD 1.62) and -2.02 (SD 1.36), respectively. **Figure 2** illustrates the longitudinal changes in BMD value and Z score at baseline and follow-up in these patients.

### Multilevel Mixed Model Analysis With BMD Z Score as the Outcome

It can be seen from the covariance parameter estimate of the null model and the results of the Z test that the dependent variable is not independent, and the measured values for a given individual are similar (Z=2.74, P=003). The ICC was 73%; that is, 73% of the variation in BMD Z score was caused by variation at the individual level. Therefore, a multilevel mixed effect model was adopted for the analysis. In the multilevel mixed-effects model with BMD Z score as an outcome, after controlling for potential confounders, cumulative GC exposure, serum levels of calcium (decreased vs. normal), phosphorus (decreased vs. normal), 25(OH)-vitamin D (deficiency vs. insufficiency) and CK were not statistically significant predictors of the BMD Z score; only age (β=-0.358, p=0.0003) and FMS (β=-0.454, p=0.031) were associated with a decrease in BMD Z score (**Table 2**).

**Table 2:** Multilevel mixed model of lumbar BMD Z score in patients with DMD.

Predictors	β (95%CI)	P value
Constant	2.118 (0.395,3.841)	0.018
Cumulative GC exposure (g)	0.0006 (-0,038,0.039)	0.974
FMS (FMS=1 vs. FMS>1)	-0.454 (-0.866,-0.043)	0.031
Calcium (normal vs. decreased, mmol/L)	0.443 (-0.697,1.584)	0.434
Phosphorus (normal vs. decreased, mmol/L)	-0.213 (-0.588,0.161)	0.255
Creatinine kinase (x10^3^ U/L)	0.013 (-0.019,0.046)	0.408
Age (year)	-0.358 (-0.537,-0.178)	<0.001
25(OH)-Vitamin D (deficiency vs. insufficiency, ng/dL)	-0.077 (-0.404,0.248)	0.630

CI, confidence interval; FMS, functional activity score.

## Discussion

Previous studies have confirmed the value of QCT in assessing BMD in pediatric patients (24–26). In DMD patients, obtaining accurate measurements of BMD is usually difficult. The most common causes for this difficulty are severe spinal rotation, scoliosis, and other musculoskeletal changes, which are present in 70%-90% of patients with advanced DMD (27). Nevertheless, QCT software can directly correct for scoliosis curves and accurately measure trabecular bones. Therefore, this study is significant because it is the first study to explore the factors associated with lumbar BMD loss in boys with DMD using QCT data.

We observed that lumbar BMD decreased longitudinally in GC-treated boys with DMD. Up to 31.6% (6/19) of patients had lumbar BMD Z scores ≤ −2 during follow-up. Suthar et al. (28) reported a reduction in BMD measured with DXA (height adjusted Z score ≤ −2) in 57% of boys with DMD in North India, which was greater than that observed in our study. Aparicio et al. (29) found osteopenia in the lumbar region in 30% of DMD patients who were not using any steroids. Therefore, the discrepancies among the results of these studies may be incomparable due to differences in population characteristics or study designs.

Our findings suggest that age is an important risk factor for lumbar BMD loss (β=-0.358, p=0.0003). Older patients with DMD may have more significant bone health impairment than younger patients who have better mobility and walking ability. Age might be useful for estimating the risk of lumbar BMD loss in boys with DMD. A previous study noted that age ≥10.5 years was associated with a reduction in BMD in boys with DMD (30). Summer et al. (31) observed that the age of appendicular lean mass loss in DMD children was approximately 12 years. However, their results may not be applicable to all DMD patients because their studies were not investigating the effects of GC exposure. Our study considered the effects of hormones and obtained similar results to other studies. Therefore, early interventions in boys with DMD could prove valuable.

It is well known that GC therapy is used to maintain muscle strength and mobilization and protect cardiac and respiratory functions in DMD (32), but potential side effects and consequent toxicity related to bone health need to be taken into account. Long-term GC treatment induces severe osteoporosis, resulting in a deregulation in bone turnover (10). The effect of GC on bone is highly dose- and time-dependent. Pharmacological doses of GC induce bone loss, which becomes evident after 6–12 months of chronic use (33). Dilber et al. found a cutoff value of 2100 mg/kg for the cumulative dose, above which adverse effects on bone were expected (34). However, Van Staa et al. found that the adverse effects of oral GC on bone were related to the daily dose rather than the cumulative dose of GC (35). In our study, we did not observe any contribution of cumulative GC dose to BMD Z score decrease using multilevel mixed-effects model analysis, which could be due to the small number of patients in the study or to the younger age and higher functional level of the patients still taking GCs. On the other hand, the use of different GC regimens may impact bone health outcomes in patients with DMD (36). Crabtree et al. (37) observed that lumbar BMD in boys with DMD was not significantly different between daily and intermittent GC regimens either at baseline or over the duration of follow-up, but a higher frequency of vertebral fracture and greater linear growth impairment were found in those receiving daily GC treatment.

In our study, an association between FMS and BMD Z score was observed in multilevel mixed-effects model analysis (β=-0.454, p=0.031). A previous study also observed that a Vignos scale ≥6 for lower extremity function can predict BMD loss (30). Progressive decline in muscle function with age is frequently accompanied by a decline in BMD and bone quality, leading to increased bone fragility fractures (13). It is still unclear whether bone defects are due to a direct mechanical effect or nonmechanical factors that also contribute to poor bone status (38). A “muscle-bone interactions” model has been introduced to explain the relationship between bone and muscle in children with neuromuscular diseases (39). Physical therapy interventions may have a positive effect in preserving and improving motor function and muscle strength in boys with DMD (21, 40), whereas the effect on improving BMD needs to be further explored.

Among the clinical characteristics, serum CK, as a diagnostic marker of DMD, was significantly higher than the normal value. Furthermore, we did not observe any contribution of CK to BMD Z score loss (β=0.013, p=0.408). The serum CK levels in children with DMD usually reached a peak at the age of three and then gradually stabilized and decreased, with an average annual decrease of 8.7%-20% (41). The changes in serum CK reflect the degree of muscle damage rather than bone damage.

Vitamin D is essential for skeletal health. It mediates the mineralization of newly synthesized osteoid tissue within bone. In the present study, we observed that the levels of serum vitamin D were low in our subjects, and vitamin D deficiency was found in 68.42% of the group at the 2-year follow-up, which can lead to decreased intestinal calcium absorption and even an imbalance in calcium metabolism (42). On the other hand, vitamin D deficiency stimulated PTH secretion. PTH promoted calcium release from bones to maintain calcium homeostasis, as observed in DMD patients in previous studies (10, 42). In our study, the mean levels of serum calcium and phosphorus were almost within normal ranges. Therefore, in multilevel mixed-effects model analysis, prediction of reduced BMD Z scores was not possible with the serum calcium (β=0.443, p=0.434) or phosphorus (β=-0.213, p=0.255) level.

Vitamin D deficiency also results in muscle weakness, a problem from which boys with DMD already suffer. Therefore, it is essential to ensure adequate daily intake of calcium and vitamin D in DMD patients even if the patients do not show increased levels of PTH or bone markers. In our analysis, we did not observe any contribution of vitamin D deficiency (vs. insufficiency) to BMD Z score loss (β=-0.077, p=0.630). This was probably due to the routine use of dietary calcium and vitamin D supplements in patients with DMD. Recent studies have shown beneficial effects of vitamin D therapy on bone health in DMD patients (42, 43). Cholecalciferol plus adequate dietary calcium intake seems to be an effective first-line approach that controls bone turnover, corrects vitamin D deficiency, and increases BMC and BMD in most patients with DMD.

The strength of this study is that mixed-effects model analysis is a valid statistical method for assessing changes in BMD over time, both within a patient and between patients. Since the data in this study are hierarchical and the measurement results for dependent variables within individuals are correlated, the multilevel model can obtain more accurate parameter estimates than the traditional linear regression model (44). Another advantage of this study is that lumbar BMD was obtained by QCT, which is more accurate than the DXA method. However, there is a lack of comparable data, as QCT equipment is not widely available. Our study has several limitations. First, a main limitation of this study is the small number of participants. Thus, some of our analyses may have been underpowered. Second, QCT has a higher radiation dose than DXA, which might limit its applicability to clinical use. Third, this retrospective study used demographic and clinical data extracted from medical records in a single institution. We are unable to assess the condition of fractures because the data on long bone and vertebral fractures are incomplete. Similarly, some data that may affect BMD were not included in the analysis, such as nutrition status, vitamin D treatment and testosterone therapy.

In conclusion, our study suggests that in DMD patients, BMD showed a gradual and progressive decreasing trend. Age and muscle function are the main contributors to lumbar BMD loss in boys with DMD. Early recognition of BMD changes may help in developing strategies for optimizing bone health, especially in patients with the risk factors identified in this study.

## Data Availability Statement

The original contributions presented in the study are included in the article/supplementary material. Further inquiries can be directed to the corresponding author.

## Ethics Statement

The studies involving human participants were reviewed and approved by the Institutional Review Board of West China Second University Hospital. Written informed consent to participate in this study was provided by the participants’ legal guardian/next of kin.

## Author Contributions

CL and H-BQ designed the study. CL, D-DY, X-GL, and WL collected the data. CL and LZ analyzed the data. YL, F-LJ, X-JC, and GN gave critical comments on the writing. CL wrote and prepared the original draft. H-BQ supervised the study. All authors contributed to the article and approved the submitted version.

## Funding

This work was funded by the Cadre Health Care Project in Sichuan Province, China: grant code 2021-1705.

## Conflict of Interest

The authors declare that the research was conducted in the absence of any commercial or financial relationships that could be construed as a potential conflict of interest.

## Publisher’s Note

All claims expressed in this article are solely those of the authors and do not necessarily represent those of their affiliated organizations, or those of the publisher, the editors and the reviewers. Any product that may be evaluated in this article, or claim that may be made by its manufacturer, is not guaranteed or endorsed by the publisher.
